# DS-7080a, a Selective Anti-ROBO4 Antibody, Shows Anti-Angiogenic Efficacy with Distinctly Different Profiles from Anti-VEGF Agents

**DOI:** 10.1167/tvst.9.9.7

**Published:** 2020-08-05

**Authors:** Yoshitaka Isumi, Shinko Hayashi, Tatsuya Inoue, Yasushi Yoshigae, Toshiyuki Sato, Jun Hasegawa, Toshinori Agatsuma

**Affiliations:** 1 Oncology Research Laboratories I, Oncology Function, R&D Division, Daiichi Sankyo Co., Ltd., Tokyo, Japan; 2 Specialty Medicine Research Laboratories I, Research Function, R&D Division, Daiichi Sankyo Co., Ltd., Tokyo, Japan; 3 Research Planning Group, Research Function, R&D Division, Daiichi Sankyo Co., Ltd., Tokyo, Japan; 4 Specialty Medicine Research Laboratories II, Research Function, R&D Division, Daiichi Sankyo Co., Ltd., Tokyo, Japan; 5 Modality Research Laboratories, Biologics Division, Daiichi Sankyo Co., Ltd., Tokyo, Japan

**Keywords:** ROBO4, antibody, choroidal neovascularization, vascular endothelial growth factor, age-related macular degeneration

## Abstract

**Purpose:**

Neovascular age-related macular degeneration (nAMD) results from choroidal neovascularization (CNV) and causes severe vision loss. Intravitreal anti-vascular endothelial growth factor (VEGF) therapies have significantly improved therapeutic outcomes; however, a substantial number of patients experience disease progression. Roundabout 4 (ROBO4) has been reported to be a vascular-specific protein that stabilizes vasculature in ocular pathological angiogenesis. To explore ROBO4 targeting as a novel treatment against neovascularization, we generated a humanized anti-human ROBO4 antibody, DS-7080a, and evaluated its efficacy.

**Methods:**

*ROBO4* mRNA in human whole eye cross-sections was examined by in situ hybridization. Human umbilical vein endothelial cell (HUVEC) migration was measured in the presence of VEGF, basic fibroblast growth factor (bFGF), hepatocyte growth factor (HGF), or conditioned medium of primary human retinal pigment epithelial (HRPE) cells. CNV was induced in cynomolgus monkeys by laser irradiation. Vascular leakage was measured by fluorescein angiography, and pathological changes were determined by histology.

**Results:**

*ROBO4* mRNA was detected in choroidal vessels of nAMD patients. DS-7080a suppressed HGF- or bFGF-induced HUVEC migration in addition to that induced by VEGF. Further, HUVEC migration induced by HRPE-conditioned medium was inhibited by either DS-7080a or ranibizumab in a similar manner, and the combination of these showed further inhibition. In a laser-induced CNV monkey model, single intravitreous administration of 1.1 mg/eye of DS-7080a reduced the incidence of grade 4 leakage from 44.45% in control eyes to 1.85% (*P* < 0.05 by Dunnett's test).

**Conclusions:**

Anti-ROBO4 antibody DS-7080a suppressed HUVEC migration in a distinctly different fashion from anti-VEGF agents and improved laser-induced CNV in non-human primates.

**Translational Relevance:**

DS-7080a may be a novel treatment option for nAMD.

## Introduction

Neovascular age-related macular degeneration (nAMD) is an ocular disease that causes vision loss, and the number of patients with this disease is increasing worldwide, especially among the elderly.^1^ Neovascular AMD results from neovascularization that develops from choroidal vessels and reaches into the subretinal or retinal lesion through Bruch's membrane.^2^ Neovascular AMD is the leading cause of severe vision loss within a short matter of time; therefore, treatments for neovascularization should be urgently provided to patients with nAMD.^3^ Intravitreal therapeutic agents that neutralize vascular endothelial growth factor (VEGF), such as ranibizumab (Lucentis) and aflibercept (Eylea), were demonstrated to reduce vascular leakage and improve visual acuity.^4^^–^^8^ Although these anti-VEGF agents have been the standard therapy for nAMD, insufficient response or refractoriness to therapy is frequently seen in patients who are treated with ranibizumab or aflibercept.^9^ Of patients treated with ranibizumab in the Minimally Classic/Occult Trial of the Anti-VEGF Antibody Ranibizumab in the Treatment of Neovascular Age-Related Macular Degeneration (MARINA) study, only 42.1% (0.5-mg injection group) of patients had better than 20/40 vision.^9^ Furthermore, the progression of nAMD can be associated with several factors other than VEGF that induce angiogenesis and vascular leakage, including inflammatory cytokines and growth factors.^10^^,^^11^ Consequently, there are unmet medical needs in the treatment of nAMD at present, and development of a therapeutic drug that has a new mechanism of action is required for nAMD therapy.

Roundabout 4 (ROBO4) is a transmembrane protein and a member of the ROBO family of receptors that are known to be axon guidance molecules.^12^ Among the four members of the ROBO family, ROBO4 is specifically expressed in vascular endothelial cells^13^^,^^14^ and plays important roles in negative regulation of vascular development, angiogenesis, and pathological neovascularization in the retina^15^ and tumor.^16^^,^^17^ However, few reports are available on ROBO4 expression in the retinal and choroidal vasculatures of human diseased eyes. ROBO4-deficient mice show enhanced ocular permeability and revascularization when subjected to oxygen-induced retinopathy, which indicates that ROBO4 plays a role in stabilizing vasculature in ocular pathological conditions.^15^^,^^18^ Slit homolog 2 (SLIT2) is a ligand for ROBO receptors regulating axonal growth, and SLIT2–ROBO4 signaling inhibits the VEGF-induced migration, tube formation, and permeability of endothelial cells in vitro and suppresses neovascularization and vascular leakage in the mouse models of retinal neovascularization and choroidal neovascularization (CNV).^15^^,^^18^^,^^19^ SLIT2–ROBO4 signaling also reduces vascular leakage caused by multiple inflammatory stimuli.^20^^–^^22^ Furthermore, it was reported that unc-5 netrin receptor B (UNC5B)^18^^,^^23^ and annexin A2^24^ are related to the anti-angiogenic effect of ROBO4. These roles of ROBO4 signaling in the regulation of vascular stability and maintenance of the vascular barrier have been expected to be a novel target for treatment of pathological neovascularization and vascular leakage such as nAMD.

In this study, we generated DS-7080a, a novel anti-ROBO4-specific humanized monoclonal immunoglobulin G2 (IgG2) antibody, and evaluated its effects on human umbilical vein endothelial cell (HUVEC) migration and a laser-induced CNV model in cynomolgus monkeys. We identified unique characteristics of the anti-ROBO4 antibody that has a different mode of action than the anti-VEGF agent ranibizumab.

## Materials and Methods

All research procedures described in this work adhered to the tenets of the Declaration of Helsinki.

### Animals

All animal procedures were performed following the ARVO Statement for the Use of Animals in Ophthalmic and Vision Research and were overseen by the Institutional Animal Care and Use Committee of Daiichi Sankyo Co., Ltd. BALB/c mice were obtained from Charles River Laboratories Japan (Yokohama, Japan). Male, 3- to 4-year-old cynomolgus monkeys (body weight, 2.95–4.15 kg) were obtained from Primate Quality Control Center, Ina Research Philippines, Inc. (Muntinlupa City, Philippines).

### Cells

Mouse myeloma SP2/0-ag14 cells (American Type Culture Collection, Manassas, VA) were cultured in Dulbecco's Modified Eagle Medium (Thermo Fisher Scientific, Waltham, MA) containing 15% fetal bovine serum (FBS), penicillin/streptomycin, and 8-azaguanine. HUVECs (Kurabo, Osaka, Japan) and human primary retinal pigment epithelial (HRPE) cells (Lonza Group, Basel, Switzerland) were cultured in HuMedia-EG2 medium (Kurabo) and retinal pigment epithelial cell growth medium (RtEGM; Lonza Group) containing 2% FBS, respectively. All cells were incubated at 37°C in a humidified atmosphere of 5% CO_2_.

To obtain the conditioned medium from polarized HRPE cells, the HRPE cells were plated on an insert with a 0.4-µm pore polyester membrane in 12-well transwell plates (Corning Inc., Corning, NY) and cultured in RtEGM supplemented with 2% FBS overnight. Subsequently, the HRPE cells were cultured in serum-free RtEGM for 34 days, during which the medium was changed every 2 or 3 days in order to polarize. The polarized HRPE cells were further cultured in serum-free HuMedia-EB2 medium (Kurabo) supplemented with 0.1% bovine serum albumin (BSA) for 1 day, and then the conditioned medium was collected from the basolateral side of the cells. To determine concentrations of 11 kinds of growth factors and nine kinds of chemokines in the conditioned medium from the polarized HRPE cells, a multiplex immunoassay (Thermo Fisher Scientific) was conducted according to the instruction provided with the Bio-Plex 200 System (Bio-Rad, Hercules, CA).

### Antibody

Human IgG2 (hIgG2) was obtained from Sigma-Aldrich (St. Louis, MO). Human IgG Fab fragment (hFab) was obtained from Jackson ImmunoResearch Laboratories (West Grove, PA). Ranibizumab was obtained from Novartis Pharmaceuticals (Basel, Switzerland).

### Antibody Generation

Six-week-old female BALB/c mice were immunized with recombinant human ROBO4 extracellular domain, which had been prepared in-house, and hybridomas were established by electrical cell fusion of the splenocytes or lymph node cells from immunized mice and mouse myeloma SP2/0-ag14 cells. The mouse monoclonal antibodies derived from the hybridomas were screened for binding to human, cynomolgus monkey, rabbit, rat, and mouse ROBO4-expressing cells, as well as for non-binding to human ROBO1, ROBO2, and ROBO3 and inhibitory activity against HUVEC migration. The constant region of the selected mouse monoclonal antibody was exchanged to human IgG2, and then this chimeric antibody was humanized by CDR grafting^25^ to obtain DS-7080a. DS-7080a was generated with mammalian cell transfection. Highly purified DS-7080a, which contained monomer fractions of >99% purity and <0.1 endotoxin units (EU)/mg, was used for the experiments. By using surface plasmon resonance, DS-7080a binds to human ROBO4 with a dissociation constant (*K*_D_) of 3.9 nM. The measured *K*_off_ was 6.6 × 10^–3^ s^–1^, and the calculated *K*_on_ was 1.7 × 10^6^ M^–1^ s^–1^.

### Human Eye Specimens

National Disease Research Interchange (NDRI, Philadelphia, PA) obtains informed consent from subjects, and this study was approved by the Daiichi Sankyo Ethical Committee. We used formalin-fixed and paraffin-embedded (FFPE) cross-sections of two human whole eyes (through macula) from one AMD donor (75 years old, female) and one normal donor (84 years old, male). The postmortem time was between 14 and 24 hours. Formalin fixation and sectioning of whole gloves were performed by an ocular histopathologist at NDRI. Slides of the indicated regions were also cut in 5-µm sections on electrostatically charged slides at NDRI. We obtained 10 slides per one donor from NDRI through the Human and Animal Bridging Research Organization (Chiba, Japan) and then subjected them to in situ hybridization analysis for assessment of ROBO4 expression.

### In Situ Hybridization

A 1009-bp DNA fragment corresponding to nucleotide positions 722 to 1730 of human ROBO4 (GenBank accession number: NM_019055) was used to generate sense or anti-sense RNA probes, and those probes were labeled with digoxigenin. Hybridization of the tissue sections with the probes was conducted based on standard procedures,^26^ and the probes were detected with Anti-Digoxigenin–AP conjugate (Roche, Basel, Switzerland) and NBT/BCIP solution (Sigma-Aldrich).

### Immunohistochemistry

For immunohistochemistry (IHC), tissue sections of monkey eyes were de-paraffined with xylene and rehydrated through an ethanol series and phosphate-buffered saline (PBS). Antigen retrieval was performed by microwave treatment with ethylenediaminetetraacetic acid (EDTA)/Tris buffer, pH9. Endogenous peroxidase was blocked with 0.3% H_2_O_2_ in methanol for 30 minutes, followed by incubation with Protein Block (Dako Pathology Solutions, Agilent, Santa Clara, CA) and avidin/biotin blocking kit (Vector Laboratories, Burlingame, CA). The sections were incubated with anti-CD31 mouse monoclonal antibody (Dako Pathology Solutions) at 4°C overnight, then incubated with biotin-conjugated rabbit anti-mouse Ig (Dako Pathology Solutions), for 30 minutes at room temperature followed by the addition of peroxidase conjugated streptavidin (Nichirei, Tokyo, Japan) for 5 minutes. Peroxidase activity was visualized by diaminobenzidine. The sections were counterstained with Mayer's Hematoxylin Solution (Muto Pure Chemicals, Tokyo, Japan), dehydrated, and then mounted with Malinol (Muto Pure Chemicals).

### HUVEC Migration Assay

HUVEC migration was evaluated in a Boyden chamber assay. HUVECs were starved in serum-free HuMedia-EB2 supplemented with 0.1% BSA for 1 day, then seeded at a density of 2 × 10^4^ cells/well into 3-µm-pore Corning FluoroBlok 96-well inserts coated with 0.1% gelatin and incubated for 5 minutes at 37°C. Lower wells were filled with 200 µL of serum-free medium supplemented with each test antibody and 10 ng/mL of recombinant human VEGF_165_ (PeproTech, Rocky Hill, NJ), 10 ng/mL of recombinant human basic fibroblast growth factor (bFGF; Corning), or 50 ng/mL of recombinant human hepatocyte growth factor (HGF; R&D Systems, Minneapolis, MN). Alternatively, some lower chambers were filled with the diluted conditioned medium from HRPE cells. The upper and lower chambers were assembled and incubated for 3 hours at 37°C. HUVECs migrating to the underside of the insert membrane were labeled by incubating with 4 µg/mL of calcein AM (Thermo Fisher Scientific) for 15 minutes at 37°C. Fluorescence intensity at 9 points/well was measured at 485-nm excitation/538-nm emission wavelengths using a SpectraMax M3 (Molecular Devices, San Jose, CA).

### Cynomolgus Monkey Laser-Induced Choroidal Neovascularization Model

A cynomolgus monkey model of CNV was generated as described previously.^27^ Briefly, under anesthesia, the Bruch's membrane in the macular region was damaged using a laser irradiator (500 mW; spot size, 50 µm; 0.1 second; 9 spots/eye) (Iridex Corporation, Mountain View, CA) with mydriatic treatment. Eight days after the laser irradiation, vehicle; 0.044, 0.22, or 1.1 mg/eye of DS-7080a; or 0.5 mg/eye of ranibizumab were intravitreously administered bilaterally to three monkeys with an injection volume of 50 µL/eye (*n* = 6 eyes for each treatment group). The highest dose of 1.1 mg/eye DS-7080a was selected because that dose is almost equivalent to 0.5 mg/eye of ranibizumab, a standard agent in this field. Also, 21 days after intravitreal dosing of 1.1 mg/eye DS-7080a, its intravitreal concentration is 100 times as high as an in vitro effective concentration of 0.125 µg/mL to inhibit HUVEC migration and is sufficient for a proof-of-concept study. RINDERON-A Ointment (Shionogi, Osaka, Japan) was applied on the conjunctiva to prevent infection.

After regular fundus photography, 20 mg/kg of a fluorescein contrast agent (FLUORESCITE Injection, 500 mg; Alcon Japan, Tokyo, Japan) was intravenously administered, and then fluorescein fundus angiography (FA) was performed under mydriasis at 7 minutes post-dosing with a contrast agent on the indicated days. FA was scored by a blinded method according to the following grading scale for CNV: grade 1, no hyperfluorescence; grade 2, hyperfluorescence without leakage; grade 3, hyperfluorescence early or mid-transit and late leakage; and grade 4, bright hyperfluorescence early or mid-transit with late leakage extending beyond the borders of the laser spot. At the end of the study, the retina and choroid membrane of the left eyes from two animals treated with vehicle and 1.1 mg/eye of DS-7080a were fixed in a tissue fixative (GenoStaff, Tokyo, Japan) for histopathology. Approximately 100 sections of 10-µm thickness that included macular region, were obtained from each eye for IHC analysis. Endotoxin levels of DS-7080a solution and vehicle used in this study were <0.1 EU/mg and <0.1 EU/mL, respectively.

### Statistical Analysis

For the monkey analysis, the difference in the incidence of grade 4 leakage on day 21 was analyzed using Dunnett's test (parametric).

## Results

### ROBO4 mRNA Is Expressed in Choroidal Vascular Endothelial-Like Cells in Patients with AMD

**Figure 1.**
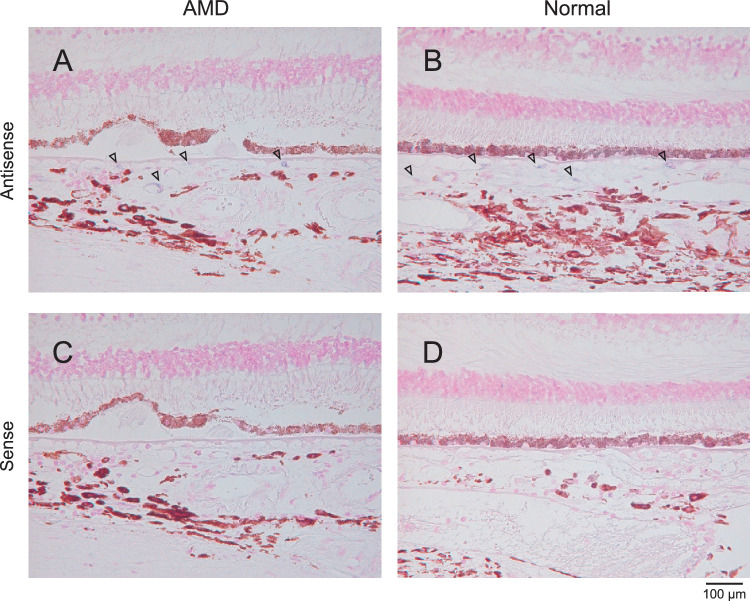
*ROBO4* mRNA is detected in endothelial-like cells of the choroidal region from patients with nAMD and healthy donors. *ROBO4* mRNA expression in choroidal vascular endothelial-like cells was determined by in situ hybridization analysis using antisense RNA probe (A, B) or sense RNA probe (C, D) in FFPE eye specimens from a nAMD patient (A, C) and a normal subject (C, F). The *arrows* indicate *ROBO4* mRNA expression in the endothelial-like cells of choroidal vessels. Magnification, 200×.

It has been reported that ROBO4 is specifically expressed in vascular endothelial cells in tumors^28^^,^^29^; however, ROBO4 expression in the choroidal vascular endothelial cells of nAMD patients and other retinal disease conditions has not yet been well characterized. To confirm ROBO4 expression in eyes from patients with AMD, we investigated *ROBO4* mRNA expression in choroidal vascular endothelial-like cells of two FFPE human eyes by in situ hybridization analysis. In the normal eye sample, *ROBO4* mRNA was expressed in choroidal vascular endothelial-like cells (Fig. 1B). Even in the eye sample from the patient with AMD, *ROBO4* mRNA was detected in choroidal vascular endothelial-like cells (Fig. 1A). Control slides for in situ hybridization using a sense probe for ROBO4 were negative for specific staining (Figs. 1C, 1D). These results indicate that *ROBO4* is actually expressed not only in normal tissues but also in the vascular lesion of AMD and could be a target molecule for developing an anti-angiogenic/vascular protective agent for the treatment of AMD.

### Anti-ROBO4-Specific Antibody, DS-7080a, Suppresses HUVEC Migration with Distinctly Different Roles Compared to Anti-VEGF Agents

**Figure 2.**
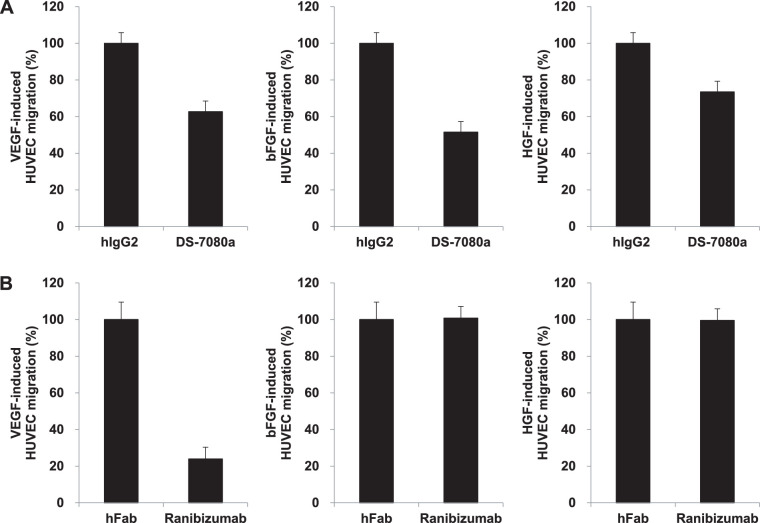
DS-7080a suppresses HUVEC migration induced by various angiogenic factors. HUVEC migration (3 hours) was determined in the presence of various angiogenic factors and is shown as a percentage relative to the control. (A) Inhibitory activity of DS-7080a (2 µg/mL, 13.7 nM) against HUVEC migration stimulated with 10 ng/mL of VEGF (left), 10 ng/mL of bFGF (middle), or 50 ng/mL of HGF (right) was evaluated. (B) Effects of ranibizumab (0.663 µg/mL, 13.7 nM) on HUVEC migration in the presence of 10 ng/mL of VEGF (left), 10 ng/mL of bFGF (middle), or 50 ng/mL of HGF (right). Each point represents the mean ± *SE* of sextuplicate wells.

We generated mouse monoclonal anti-human ROBO4 antibodies by immunization of recombinant human ROBO4 extracellular domain. The constant region of the selected mouse monoclonal antibody was exchanged to human IgG2, and then the chimeric antibody was humanized by CDR grafting^25^ to obtain DS-7080a. DS-7080a specifically bound to ROBO4, but not to other ROBO family members and angiogenic factors such as VEGF, bFGF, and HGF (Supplementary Figs. S2, S5). To examine the biological effect of DS-7080a against vascular endothelial cells, the effect of DS-7080a on HUVEC migration was evaluated. DS-7080a reduced HUVEC migration stimulated with not only 10 ng/mL of VEGF but also 10 ng/mL of bFGF and 50 ng/mL of HGF (Fig. 2A). As expected, an anti-VEGF agent, ranibizumab, did not inhibit HUVEC migration induced by bFGF and HGF (Fig. 2B). DS-7080a showed dose-dependent inhibition of HUVEC migration and had maximum suppression at concentrations greater than 0.125 µg/mL (Supplementary Fig. S3). These results suggest that DS-7080a works in a different way than anti-VEGF agents and broadly suppresses endothelial cell migration stimulated with various angiogenic factors such as VEGF, bFGF, and HGF.

### DS-7080a Suppresses HUVEC Migration Induced by Conditioned Medium of Polarized HRPE Cells

**Figure 3.**
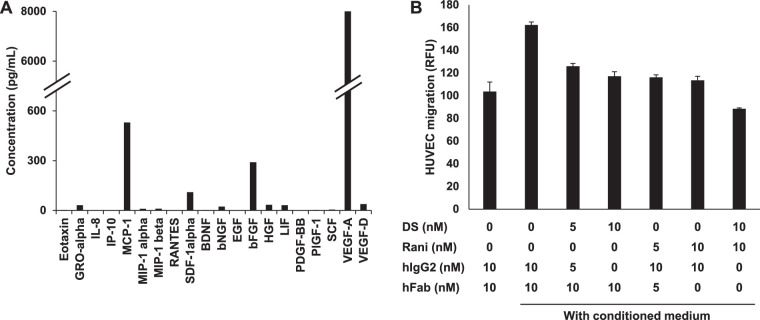
DS-7080a attenuates HUVEC migration induced by conditioned medium from human retinal pigment epithelial cells. (A) The concentrations of 11 growth factors and nine chemokines in the conditioned medium derived from polarized HRPE cells were determined by multiplex immunoassay. To obtain the conditioned medium, HRPE cells were cultured on transwells for about 1 month to allow for cell polarization. Each column is represented by the mean concentration of triplicate wells. (B) Effects of DS-7080a (DS) and ranibizumab (Rani) on HUVEC migration stimulated with the HRPE-conditioned medium were evaluated. Total contents of antibodies and Fab fragments were adjusted by adding hIgG2 and hFab. Migrated HUVECs were measured after 3 hours of incubation. HUVEC migration is shown in relative fluorescent units (RFU). Each point represents the mean ± *SE* of sextuplicate wells.

The microenvironment of disease areas is more complex, and angiogenesis may rely on multiple angiogenic factors. Angiogenic factors secreted from RPE cells are reported to play important roles in the development of CNV.^10^^,^^30^^–^^32^ Actually, in the conditioned medium from polarized HRPE cells, VEGF-A, monocyte chemoattractant protein 1 (MCP-1), bFGF, and other factors were detected (Fig. 3A). Therefore, to evaluate the effect of DS-7080a in the condition relating to multiple factors such as pathological angiogenesis, we employed HUVEC migration induced by conditioned medium from HRPE cells and examined the inhibitory activity of DS-7080a. DS-7080a reduced HRPE-induced HUVEC migration to a similar extent as ranibizumab (Fig. 3B). Furthermore, the combination of DS-7080a and ranibizumab showed an additive effect relative to the maximum effect of each single antibody treatment in this evaluation (Fig. 3B).

### Intravitreous Administration of DS-7080a to Cynomolgus Monkeys Suppresses Lesion Leakage with Histological Amelioration in CNV Induced by Laser Irradiation

**Figure 4.**
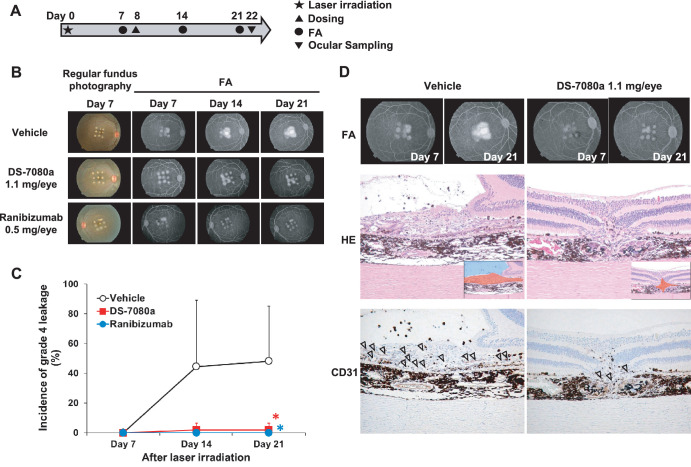
DS-7080a reduces the incidence of neovascularization in a nAMD model in cynomolgus monkeys. (A) Dose, observation, and sampling schedule of the experiments in the choroidal neovascular model. Laser irradiation was performed 8 days prior to intravitreous dosing of DS-7080a and ranibizumab (day 0). DS-7080a (0.044, 0.22, and 1.1 mg/eye), ranibizumab (0.5 mg/eye), or vehicle were administered intravitreously to six eyes of three monkeys each (*n* = 6) on day 8. FA was carried out on day 7 (pre-dosing), day 14, and day 21. (B) The regular photography and FA of cynomolgus monkeys’ eyes in the CNV model. (C) The leakage of fluorescein in the lesion of laser injury was scored based on fluorescence FA using a blinded method according to the grading scale for CNV. The percentages of grade 4 leakage (bright hyperfluorescence early or mid-transit, with late leakage extending beyond the borders of the laser spot) were calculated, and the values for vehicle, ranibizumab, and 1.1-mg/eye of DS-7080a groups were plotted. Each point represents the mean ± *SD* (*n* = 6). Parametric Dunnett's test, ^*^P < 0.05. (D) FA (days 7 and 21) of vehicle- and DS-7080a-treated eyes. Corresponding hematoxylin and eosin (H&E) and CD31 staining images at day 22 (at the end of the study) are exhibited below. The insets in the H&E staining illustrate the proliferative lesion of choroid (*red*) and fluid accumulation (*blue*). *Arrowheads* indicate CD31-positive endothelial cells.

Because DS-7080a bound to human ROBO4 and cross-reacted to cynomolgus monkey and rabbit ROBO4 orthologs, but not those of mouse and rat (Supplementary Fig. S1), we examined the efficacy of DS-7080a in a primate CNV model. The scheme of the study is illustrated in Fig. 4A. The Bruch's membranes were damaged by laser irradiation to induce CNV (day 0). On day 8 (8 days after laser irradiation), vehicle; 0.044, 0.22, or 1.1 mg/eye of DS-7080a; or 0.5 mg/eye of ranibizumab were administered to the monkeys by intravitreous injection. The degree of CNV at each laser-damaged site (nine sites/eye in total) in the choroid membrane was classified into four grades based on fluorescein extravasation images in the FA (Figs. 4B, 4D). Scoring was conducted by two independent graders (TS and MT: Mamoru Tanaka, Ina Research Inc.), and the incidence of grade 4 leakage was obtained. Between-grader analysis indicted they had high correlation (*R*^2^ = 0.99), and the scores of TS were then examined by post hoc statistical analyses to compare the vehicle and ranibizumab groups or the vehicle and 1.1-mg/eye of DS-7080a groups.

On day 7 (pre-dosing), grade 4 leakage was not observed in any group. Thereafter, in the vehicle group, the incidence of grade 4 leakage on day 14 was 44.45%; on day 21, it was 48.15% (Fig. 4C). Ranibizumab completely suppressed the development of the grade 4 leakage at a dose of 0.5 mg/eye on day 21 following its administration (*P* < 0.05). Notably, 1.1 mg/eye of DS-7080a suppressed the incidences of grade 4 leakage to a similar extent as ranibizumab on day 21 (*P* < 0.05) (Figs. 4B, 4C). Lower doses (0.22 mg/eye and 0.044 mg/eye) of DS-7080a did not suppress the incidence of grade 4 leakage (Supplementary Fig. S4). Histological analysis of the spots damaged by laser irradiation was performed using day 21 samples. Treatment with 1.1 mg/eye of DS-7080a reduced the proliferative lesion of choroid and fluid accumulation within the retina relative to vehicle treatment (Fig. 4D). In addition, the number of cluster of differentiation 31 (CD31)-positive endothelial cells decreased after treatment with a high dose of DS-7080a (Fig. 4D), and this is considered to be one of the mechanisms of DS-7080a to prevent CNV. These results demonstrate that DS-7080a inhibits neovascularization and vascular leakage induced by laser irradiation.

## Discussion

Treatments for nAMD have developed from the emergence of anti-VEGF agents. At present, four anti-VEGF agents—pegaptanib sodium (Macugen), ranibizumab (Lucentis), bevacizumab (Avastin, off-label use), and aflibercept (Eylea)—have been prescribed to patients with nAMD along with brolucizumab (Beovu), which was recently approved by the US Food and Drug Administration. Ranibizumab has been widely used for nAMD therapy since its approval in 2006. The visual acuity of approximately 40% of patients treated with ranibizumab improved to 20/40 vision or better in the MARINA study, but about 10% of patients showed visual acuity worse than 20/200 in spite of treatment with ranibizumab.^4^ Aflibercept, which was approved after ranibizumab, neutralizes not only VEGF-A but also VEGF-B and placenta growth factor (PlGF) with high affinity unlike other anti-VEGF agents. Similar to ranibizumab, nearly 40% of patients treated with aflibercept did not achieve improvement of more than 5 in their Early Treatment Diabetic Retinopathy Study (EDTRS) letter score.^7^ To improve the efficacy of these anti-VEGF agents, switching from one anti-VEGF agent to another has been evaluated in various clinical studies. The effectiveness of switching therapies has been determined via anatomic analyses such as retinal thickness, but improvements in visual acuity have not been observed.^33^ Therefore, treatments other than anti-VEGF agents are required for the treatment of nAMD.^11^ It is important to understand the resistance mechanism to anti-VEGF therapy, and multiple mechanisms are thought to be involved. One such mechanism is tachyphylaxis, caused by VEGF upregulation and/or development of anti-drug antibodies, which reduces the efficacy of anti-VEGF therapeutics.^34^ Another mechanism is the contribution of other angiogenic factors that lead to vascular leakage and development of CNV, such as growth factors and chemokines, among others.^10^^,^^11^^,^^31^^,^^32^^,^^35^

Involvement of ROBO4, an endothelial-specific transmembrane receptor, in angiogenesis has been reported in published literature, although the anti-angiogenic mechanism of the ROBO4 axis has not been fully characterized. SLIT2 attenuates angiogenesis and vascular leakage induced by not only VEGF but also inflammatory stimuli, and the effects are ROBO4 dependent.^15^^,^^20^^,^^21^ In addition, hyperpermeability observed in ROBO4-deficient mice and endothelial cells indicates that ROBO4 stabilizes vasculature.^18^^,^^36^ Another SLIT2-independent mechanism was also reported, indicating that ROBO4 stabilizes bloods vessels via another endothelial guidance receptor, unc-5 netrin receptor B (UNC5B), a downstream inhibitory molecule against VEGF.^18^^,^^23^ In addition, it was recently reported that annexin A2 decreased trans-endothelial permeability via ROBO4 signaling.^24^ Furthermore, we confirmed the expression of *ROBO4* in the choroidal vascular endothelial-like cells from patients with ocular disease, including nAMD (Fig. 1). We suggest that ROBO4 could become a target molecule for a broad-spectrum therapeutic agent for nAMD that is distinct from the current standard of anti-VEGF monotherapies.

In this study, we obtained DS-7080a, a humanized anti-ROBO4 specific monoclonal IgG2 antibody that inhibits VEGF-induced HUVEC migration, and observed maximum suppression at concentrations greater than 0.125 µg/mL (0.857 nM), comparable to that of ranibizumab reported in the literature (Supplementary Fig. S3).^37^ DS-7080a also inhibited VEGF-independent HUVEC migration induced by multiple bFGF, HGF, and HRPE-derived factors (Figs. 2, 3), indicating that DS-7080a induced anti-angiogenesis downstream of VEGF, HGF, bFGF, and possibly other factors via a mechanism other than VEGF signaling. Because non-VEGF growth factors are reported to be associated with nAMD,^11^^,^^32^ the effect of DS-7080a on nAMD may be expected, especially in patients refractory to the current therapy. The effect of DS-7080a was comparable to that of ranibizumab in HUVEC migration induced by multiple HRPE-derived factors and laser-induced CNV in a primate disease model (Figs. 3B, 4). Interestingly, we showed the additive effect of DS-7080a in combination with ranibizumab (Fig. 3B). This result further indicates that regulation between DS-7080a and anti-VEGF agents is independent of each other, and a combination therapy of these two agents may be more effective than monotherapy of each single agent.

Although its mechanism is unclear, DS-7080a possibly mediates signaling via common downstream molecules of angiogenesis, such as Src and Rac1, reported as a SLIT2–ROBO4 signal,^15^^,^^19^ and further elucidation is needed to understand DS-7080a–ROBO4-mediated anti-angiogenesis. In a nAMD model in cynomolgus monkeys, the incidence of grade 4 leakage was decreased in eyes with 1.1-mg/eye DS-7080a treatment, but not by 0.22-mg/eye treatment. Tissue drug concentrations may be the reason. We measured DS-7080a levels in vitreous humor and serum in this study. DS-7080a levels in vitreous humor on day 21 or day 22 (*n* = 6 eyes) were 0.132 ± 0.129 µg/mL in the 0.22-mg/eye group and 0.268 ± 0.272 µg/mL in the 1.1-mg/eye group. Serum levels on day 21 (*n* = 3) were 0.016 ± 0.0183 µg/mL in the 0.22-mg/eye group and 1.360 ± 0.927 µg/mL in the 1.1-mg/eye group (data not shown). Thus, only serum levels after the 1.1-mg/eye DS-7080a treatment were sufficient to show efficacy (i.e., about 10 times as high as its in vitro effective concentration of 0.125 µg/mL) (Fig. 4C; Supplementary Figs. S3, S4). This is reasonable, as ROBO4 is expressed specifically in vascular endothelial cells; thus, drug concentrations in serum could explain the pharmacokinetic/pharmacodynamic relationship.

In conclusion, DS-7080a, an anti-ROBO4 humanized antibody, is a novel anti-angiogenic antibody that is expected to serve as a therapeutic agent for pathological angiogenesis and vascular leakage in the treatment of nAMD patients. To our knowledge, this is the first report where an anti-ROBO4 antibody showed inhibitory activity to angiogenesis in an in vitro/in vivo model. These results support further investigation of DS-7080a for the treatment of ocular neovascular diseases in the clinical setting.

## Supplementary Material

Supplement 1

Supplement 2

Supplement 3

Supplement 4

Supplement 5

Supplement 6
