# Supplementary material for: DS-7080a, a Selective Anti-ROBO4 Antibody, Shows Anti-Angiogenic Efficacy with Distinctly Different Profiles from Anti-VEGF Agents
Source: Transl Vis Sci Technol. 2020 Aug 5;9(9):7. doi: 10.1167/tvst.9.9.7 (PMC7442859; doi:10.1167/tvst.9.9.7)
Supplement: Supplement 1 [file tvst-9-9-7_s001.pdf]

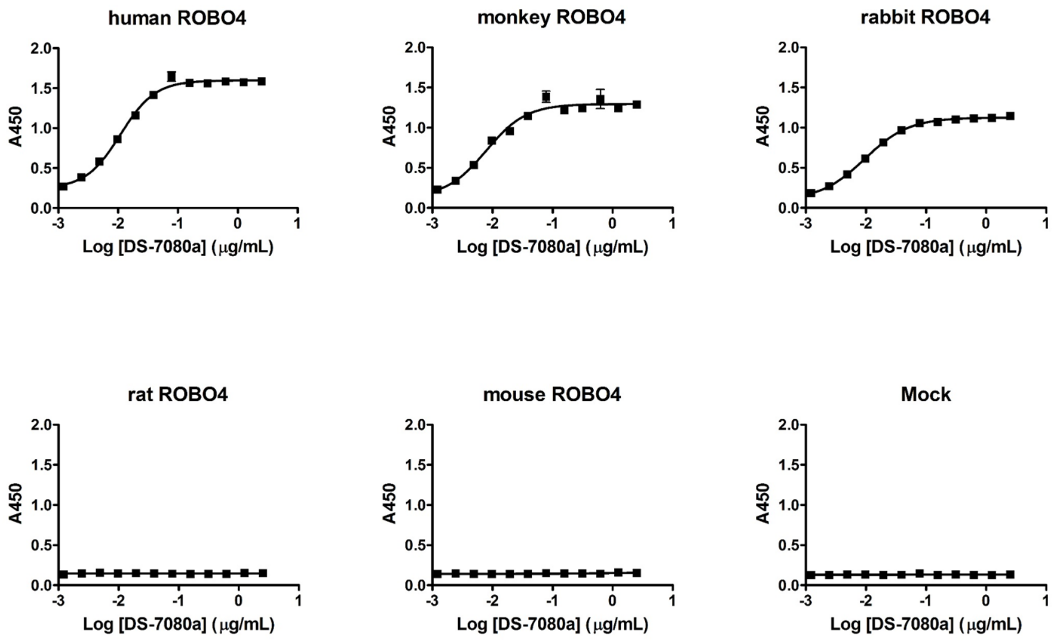

Supplementary Figure S1 DS-7080a cross-reacts to human, cynomolgus monkey, and rabbit ROBO4.

The species cross-reactivity of DS-7080a to ROBO4 orthologs was measured by cell ELISA. Each absorbance value at 450 nm (A450) represents the mean ± SE from triplicate wells.
