# Supplementary material for: DS-7080a, a Selective Anti-ROBO4 Antibody, Shows Anti-Angiogenic Efficacy with Distinctly Different Profiles from Anti-VEGF Agents
Source: Transl Vis Sci Technol. 2020 Aug 5;9(9):7. doi: 10.1167/tvst.9.9.7 (PMC7442859; doi:10.1167/tvst.9.9.7)
Supplement: Supplement 2 [file tvst-9-9-7_s002.pdf]

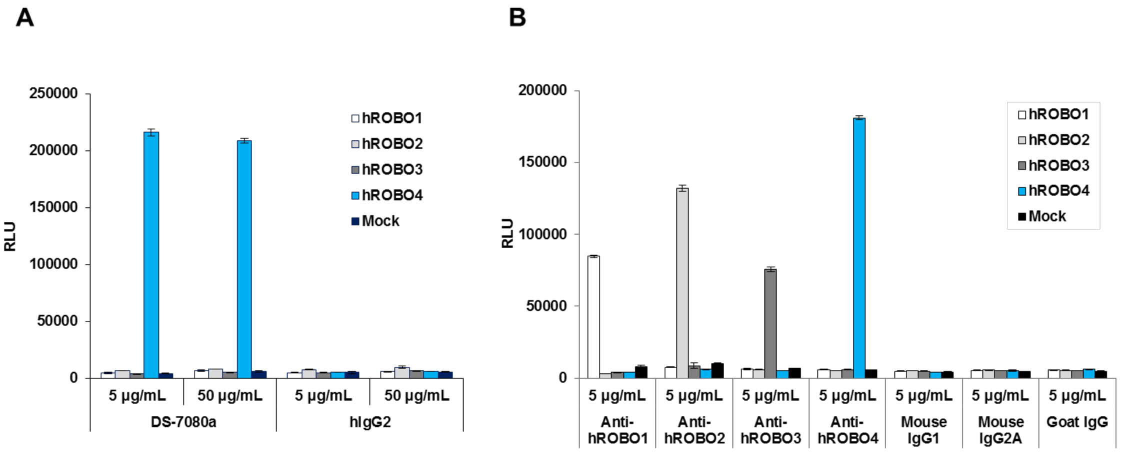

Supplementary Figure S2 DS-7080a specifically binds to ROBO4 among ROBO family proteins.

(A) The binding of DS-7080a and its isotype control, hIgG2, to each protein of ROBO family members was measured by cell ELISA. (B) To confirm expression of each protein of ROBO family members on the surface of the cells, the binding activity of the specific anti-body against each protein of the ROBO family was evaluated by cell ELISA. Mouse IgG1, mouse IgG2A, and goat IgG were used as the isotype controls (negative control). The binding was shown as luminescence intensity (relative light units, RLU), and each value represents the mean ± SE of triplicate wells.
