# Supplementary material for: DS-7080a, a Selective Anti-ROBO4 Antibody, Shows Anti-Angiogenic Efficacy with Distinctly Different Profiles from Anti-VEGF Agents
Source: Transl Vis Sci Technol. 2020 Aug 5;9(9):7. doi: 10.1167/tvst.9.9.7 (PMC7442859; doi:10.1167/tvst.9.9.7)
Supplement: Supplement 3 [file tvst-9-9-7_s003.pdf]

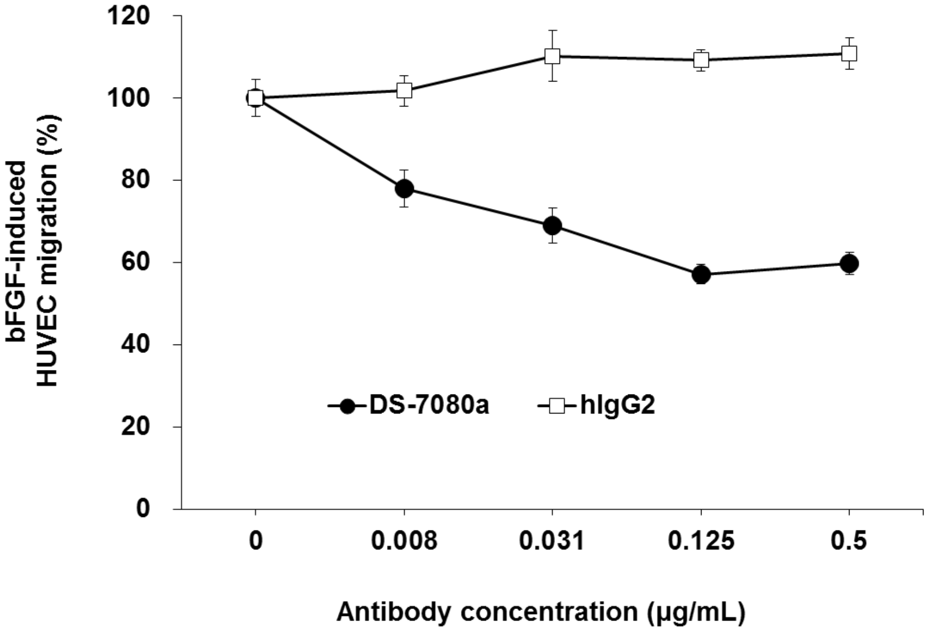

Supplementary Figure S3 DS-7080a dose-dependently suppresses HUVEC migration induced by bFGF.

The inhibitory activities of DS-7080a and hIgG2 at the concentrations ranging from 0.008 to 0.5 μg/mL against HUVEC migration stimulated with 10 ng/mL of bFGF were evaluated. Each point represents the mean ± SE of sextuplicate wells.
