# Supplementary material for: DS-7080a, a Selective Anti-ROBO4 Antibody, Shows Anti-Angiogenic Efficacy with Distinctly Different Profiles from Anti-VEGF Agents
Source: Transl Vis Sci Technol. 2020 Aug 5;9(9):7. doi: 10.1167/tvst.9.9.7 (PMC7442859; doi:10.1167/tvst.9.9.7)
Supplement: Supplement 4 [file tvst-9-9-7_s004.pdf]

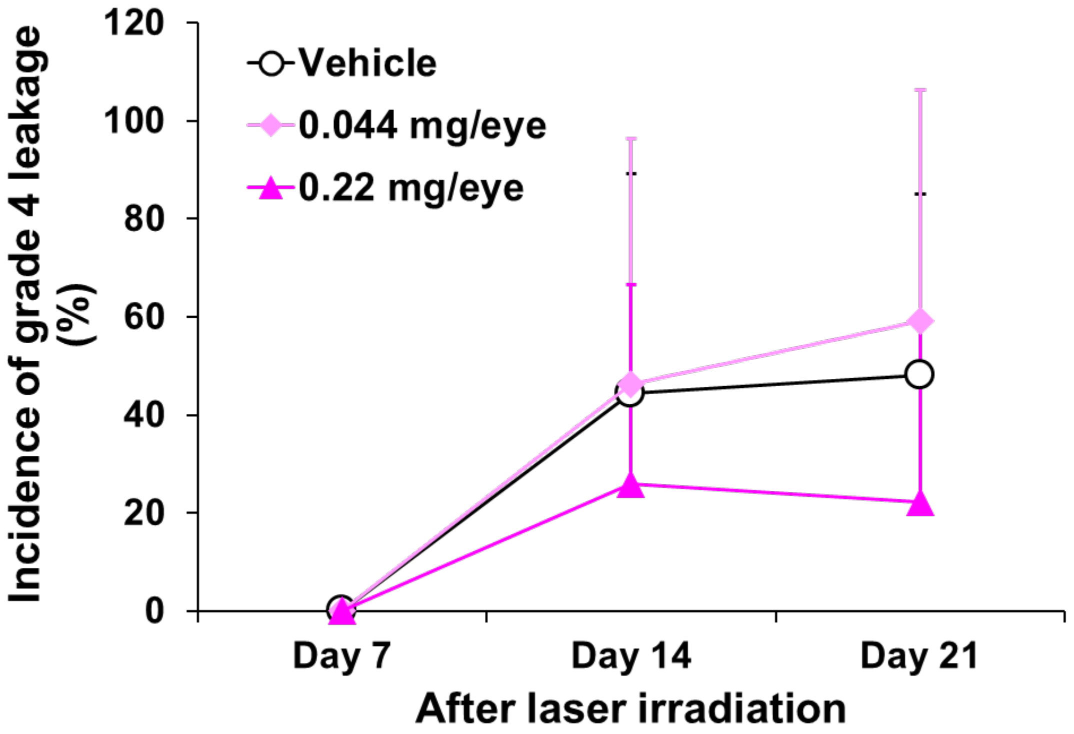

Supplementary Figure S4 Low-dose DS-7080a does not reduce the incidence of neovascularization in a nAMD model in cynomolgus monkeys.

Laser irradiation was performed 8 days prior to intravitreous dosing of DS-7080a (Day 0). 0.044 mg/eye of DS-7080a, 0.22 mg/eye of DS-7080a and vehicle in addition to 1.1 mg/eye of DS-7080a and 0.5 mg/eye of ranibizumab were administered intravitreously to 6 eyes of 3 monkeys each (n=6) on Day 8. FA was carried out on Day 7 (pre-dosing), Day 14 and Day 21. The leakage of fluorescein in the lesion of laser injury were scored based on fluorescence fundus angiography by a blinded method according to the grading scale for CNV, and the percentages of grade 4 leakage (bright hyperfluorescence early or midtransit, with late leakage extending beyond the borders of the laser spot) were calculated. Each point represents the mean + SD (n=6). Note that this figure shows the data of vehicle, 0.044 mg/eye and 0.22 mg/eye of DS-7080a treatment
