# Supplementary material for: DS-7080a, a Selective Anti-ROBO4 Antibody, Shows Anti-Angiogenic Efficacy with Distinctly Different Profiles from Anti-VEGF Agents
Source: Transl Vis Sci Technol. 2020 Aug 5;9(9):7. doi: 10.1167/tvst.9.9.7 (PMC7442859; doi:10.1167/tvst.9.9.7)
Supplement: Supplement 5 [file tvst-9-9-7_s005.pdf]

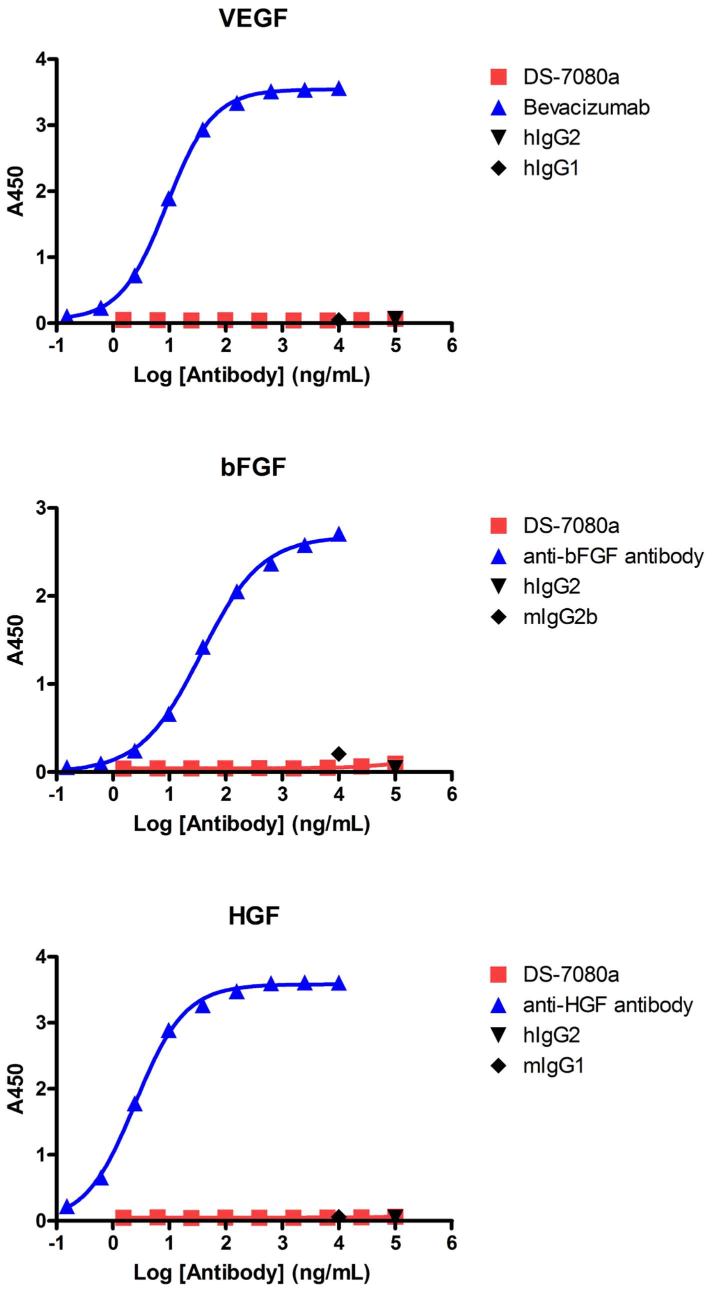

Supplementary Figure S5 DS-7080a does not bind to VEGF, bFGF and HGF.

The binding of DS-7080a, anti-VEGF antibody (bevacizumab), anti-bFGF antibody, and anti-HGF antibody to recombinant VEGF, bFGF and HGF of human origin were measured by ELISA. Human IgG2, human IgG1, mouse IgG2a and mouse IgG1 were used as the negative control. The binding was shown as the A450, and each value represents the mean ± SE of triplicate wells.
