# Supplementary material for: DS-7080a, a Selective Anti-ROBO4 Antibody, Shows Anti-Angiogenic Efficacy with Distinctly Different Profiles from Anti-VEGF Agents
Source: Transl Vis Sci Technol. 2020 Aug 5;9(9):7. doi: 10.1167/tvst.9.9.7 (PMC7442859; doi:10.1167/tvst.9.9.7)
Supplement: Supplement 6 [file tvst-9-9-7_s006.pdf]

DS-7080a, a selective anti-ROBO4 antibody, shows anti-angiogenic efficacy with distinctly different profiles from anti-VEGF agents

Yoshitaka Isumi, Shinko Hayashi, Tatsuya Inoue, Yasushi Yoshigae, Toshiyuki Sato, Jun Hasegawa, Toshinori Agatsuma

Supplementary Material

Supplementary Materials and Methods

Cell culture

COS-1 cells were obtained from DS Pharma Biomedical (Osaka, Japan) and cultured in DMEM (Thermo Fisher Scientific, Waltham, MA, USA) supplemented with 10% (v/v) heat-inactivated FBS.

Plasmid construction

The cDNAs of full-length cynomolgus monkey, rabbit, rat, and mouse *ROBO4* were obtained by PCR reaction using cynomolgus monkey kidney cDNA, Rabbit Heart cDNA (Zyagen, San Diego, CA, USA), Rat Spleen QUICK-Clone cDNA (Takara Bio, Shiga, Japan) and Mouse Heart QUICK-Clone cDNA (Takara Bio) as templates, respectively. And then, these cDNAs were inserted into the pCI vector (Promega, Madison, WI, USA) for expression on mammalian cells. In addition, the cDNAs of full-length human *ROBO1* (GenBank accession number: NM_133631), human *ROBO2* (GenBank accession number: NM_001290040), human *ROBO3* (GenBank accession number: NM_022370) and human *ROBO4* (GenBank accession number: NM_019055) were inserted into the pCI vector.

Species cross-reactivity

COS-1 cells were transfected with expression vectors for each ortholog using FuGENE6 (Promega). The transfectants were plated at a density of 4×10^4 cells/well into 96-well collagen coated plates and cultured for 1 day. Serial dilutions of DS-7080a were added to transfectants in triplicate and the plates were incubated for 1 h at 4°C. After washing, HRP-anti-human IgG (Jackson ImmunoResearch Laboratories, West Grove, PA, USA) diluted to 1:500 was added to the transfectants, and the plates were incubated for 1 h at 4°C. To detect the horseradish peroxidase activity, the TMB Microwell Peroxidase Substrate System (SeraCare, Milford, MA, USA) was used, and the absorbance at 450 nm (A450) was measured with a microplate reader, SpectraMAX M3 (Molecular Devices, San Jose, CA, USA). The binding affinities of DS-7080a to human, cynomolgus monkey, and rabbit ROBO4 were calculated as EC_{50} values with SAS System Release 9.2 (SAS Institute, Cary, NC, USA) using a Sigmoid Emax model.

Binding specificity

COS-1 cells were transfected with expression vectors for each ROBO family protein using Lipofectamine 2000 (Thermo Fisher Scientific). The transfectants were plated at a density of 4×10^4 cells/well into 96-well collagen coated black plates and cultured overnight.

DS-7080a or hIgG2 (Sigma-Aldrich, St. Louis, Mo, USA) were added to transfectants in triplicate, and the plates were incubated for 1 h at 4°C. After washing, HRP-anti-human IgG diluted to 1:1000 was added to transfectants, and the plates were incubated for 1 h at 4°C. To confirm the expression of ROBO family proteins on the surface of the cells, specific antibodies against human ROBO1 (anti-hROBO1), ROBO2 (anti-hROBO2), ROBO3 (anti-hROBO3), and ROBO4 (anti-hROBO4) and these isotype controls, mouse IgG1, mouse IgG2A, and goat IgG, were used (R&D Systems, Inc., Minneapolis, MN, USA). Binding of these antibodies were detected by HRP-conjugated anti-mouse IgG (1:500) or HRP-conjugated anti-goat IgG (1:1000) used as secondary antibodies (Jackson ImmunoResearch Laboratories). To evaluate horseradish peroxidase, SuperSignal ELISA Pico Chemiluminescent Substrate (Thermo Fisher Scientific) was used, and luminescence intensity (relative light units, RLU) was measured with SpectraMAX M3.

Binding of DS-7080a to recombinant VEGF, bFGF and HGF of human origin

Two hundred ng/mL of recombinant human VEGF165 (VEGF; PeproTech, Rocky Hill, NJ, USA), recombinant human basic FGF (bFGF, R&D Systems) and recombinant human HGF (HGF, R&D Systems) were coated overnight at 4°C onto 96-well plates. After washing, wells were blocked for 1 h at room temperature. Serial dilutions of DS-7080a, anti-VEGF antibody (bevacizumab, Genentech inc., South San Francisco, CA, USA), anti-bFGF antibody (R&D systems), anti-HGF antibody (R&D systems), isotype control human IgG2 (hIgG2, Merck KGaA, Darmstadt, Germany), isotype control human IgG1 (hIgG1, BioLegend, Inc., San Diego, CA, USA), isotype control mouse IgG2b (mIgG2b, BioLegend, Inc.) or isotype control mouse IgG1 (mIgG1, BioLegend, Inc.) were added to the wells in triplicate and the plates were incubated for 1 h at room temperature. After washing, HRP-anti-human IgG or HRP-anti-mouse IgG (Jackson ImmunoResearch Laboratories) diluted to 1:1000 was added to the wells, and the plates were incubated for 30 min at room temperature. After washing, to detect the horseradish peroxidase activity, the TMB Microwell Peroxidase Substrate System was used, and the A450 was measured with a microplate reader, SpectraMAX M3.

Supplementary Results

DS-7080a cross-reacts to cynomolgus monkey and rabbit ROBO4, but not to rodent orthologs.

The species cross-reactivity of DS-7080a to ROBO4 ortholog proteins was measured by cell ELISA (Supplementary Fig. S1). Among the ROBO4 orthologs, DS-7080a bound to human, cynomolgus monkey and rabbit ROBO4, but not to rat and mouse ROBO4. The EC_{50} values of DS-7080a to human, cynomolgus monkey and rabbit ROBO4 were 0.009 μg/mL (0.065 nM), 0.008 μg/mL (0.052 nM) and 0.01 μg/mL (0.066 nM), respectively.

DS-7080a specifically binds to ROBO4 among ROBO family proteins.

The binding of DS-7080a to each ROBO family was measured by cell ELISA (Supplementary Fig. S2A). The binding specificity of DS-7080a was determined at the concentrations of 5 μg/mL and 50 μg/mL, which are the excessive concentrations based on the EC_{50} value of DS-7080a binding to human ROBO4. DS-7080a specifically bound to human ROBO4-expressing cells, but not to ROBO1-, ROBO2-, and ROBO3-expressing cells at both concentrations. The expression of each ROBO family protein on the surface of the cells was confirmed by binding of a specific antibody against each protein to the transfected cells (Supplementary Fig. S2B).

DS-7080a suppresses bFGF-induced HUVEC migration with dose-dependency.

DS-7080a dose-dependently suppressed HUVEC migration induced by bFGF (Supplementary Fig. S3). DS-7080a had maximum suppression at concentrations of more than 0.125 μg/mL.

Low-dose DS-7080a does not reduce the incidence of neovascularization in a nAMD model in cynomolgus monkeys.

The Bruch's membranes were damaged by laser irradiation to induce CNV (Day 0). On Day 8, vehicle, 0.044 mg/eye and 0.22 mg/eye of DS-7080a in addition to 1.1 mg/eye of DS-7080a and 0.5 mg/eye of ranibizumab were administered to the monkeys by intravitreous injection. The degree of CNV at each laser-damaged site (9 sites/eye in total) in the choroid membrane was classified into 4 grades based on fluorescein extravasation images in the FA, and post-hoc statistical analyses were performed for the comparison between vehicle and 0.044 mg/eye groups or vehicle and 0.22 mg/eye of DS-7080a groups.
0.22 mg/eye and 0.044 mg/eye of DS-7080a treatment did not suppressed incidences of grade 4 leakage compared to the vehicle group (Supplementary Fig. S4).

DS-7080a does not bind to VEGF, bFGF and HGF.

The non-specific binding of DS-7080a to VEGF, bFGF and HGF was measured by ELISA-type binding assay (Supplementary Fig. S5). DS-7080a did not bind to the wells coated with VEGF, bFGF and HGF, even at a concentration of 100 μg/mL. In contrast, specific antibodies against VEGF, bFGF and HGF bound to the wells coated with each protein. The data suggest that DS-7080a did not bind to these angiogenic factors.
